# NETosis in Long-Term Type 1 Diabetes Mellitus and Its Link to Coronary Artery Disease

**DOI:** 10.3389/fimmu.2021.799539

**Published:** 2022-01-05

**Authors:** Sverre Grøver Aukrust, Kristine Bech Holte, Trine B. Opstad, Ingebjørg Seljeflot, Tore Julsrud Berg, Ragnhild Helseth

**Affiliations:** ^1^ Center for Clinical Heart Research, Department of Cardiology, Oslo University Hospital Ullevål, Oslo, Norway; ^2^ Department of Endocrinology, Oslo University Hospital Aker, Oslo, Norway; ^3^ Faculty of Medicine, University of Oslo, Oslo, Norway

**Keywords:** neutrophil extracellular traps, glucose, neutrophils, NETs, coronary artery disease, type 1 diabetes mellitus

## Abstract

**Background:**

Neutrophil extracellular traps NETs have been linked to glucose and the pathogenesis of type 1 diabetes mellitus (T1DM). NETs also play a role in vascular inflammation and the development of coronary artery disease (CAD). The role of NETs in CAD progression in patients with long-term T1DM is unclear. We aimed to 1) investigate whether levels of circulating NETs markers were elevated in long-term T1DM subjects compared to controls, and 2) explore whether levels of NETs were related to the presence of CAD.

**Material and Methods:**

102 patients with > 45 years of T1DM and 75 age-matched controls were enrolled in a cross-sectional study. Median age was 62 years. Computed tomography coronary angiography (CTCA) was performed in 148 subjects without established coronary heart disease. For the current study, CAD was defined as a coronary artery stenosis >50%. Double-stranded deoxyribonucleic acid (dsDNA) was measured by a nucleic acid stain, myeloperoxidase-DNA (MPO-DNA), citrullinated histone 3 (H3Cit) and peptidylarginine deiminase 4 (PAD4) by ELISAs, while gene expression of PAD4 was measured in leukocytes from PAXgene tubes.

**Results:**

Circulating MPO-DNA levels were significantly lower in patients with T1DM than in controls (0.17 vs 0.29 OD, p<0.001), while dsDNA, H3Cit, PAD4 and gene expression of PAD4 did not differ with respect to the presence of T1DM. There were no significant associations between NETs markers and HbA1c in the T1DM group. None of the NETs markers differed according to the presence of CAD in patients with T1DM. While all circulating NETs markers correlated significantly with circulating neutrophils in the control group (r=0.292-393, p<0.014), only H3Cit and PAD4 correlated with neutrophils in the T1DM group (r= 0.330-0.449, p ≤ 0.001).

**Conclusions:**

In this cross-sectional study of patients with long-term T1DM and age-matched controls, circulating NETs levels were not consistently associated with the presence of T1DM or glycemic status, and did not differ according to the presence of CAD in patients with T1DM. Our results entail the possibility of altered neutrophil function and reduced NETosis in T1DM. This warrants further investigation.

## Introduction

Neutrophils play a pivotal role in innate immunity by cytokine production, phagocytosis, and release of neutrophil extracellular traps (NETs) (1, 2). NETs are extruded, decondensed neutrophil chromatin and granular enzymes that form extracellular mesh-like structures upon neutrophil activation. NETs formation is often referred to as NETosis. The molecular process of NETosis is not fully understood, but crucial events are suggested to involve the production of reactive oxygen species (ROS) and decondensation of neutrophil chromatin by the enzyme peptidylarginine deiminase 4 (PAD4) (3). With cell membrane rupture, spindle-like structures of double-stranded deoxyribonucleic acid (dsDNA), histones and neutrophil proteins are released into the extracellular space as NETs (4).

There are numerous activators of NETosis, including interleukins, lipopolysaccharide, gram-positive and negative bacteria, cholesterol crystals, and fungi (3). Hyperglycemia has also been reported to initiate NETosis. This is hypothesized to be related to increase in ROS caused by effects of glucose on NADPH oxidase and the mitochondria (5–8).

Studies have shown a reduced number of circulating neutrophils in patients with prediabetes or early-onset type 1 diabetes mellitus (T1DM) (9), thought to be caused by a combination of reduced bone marrow production and increased peripheral destruction. The latter could be linked to increased NETosis (9). Some studies suggest normalization of neutrophil counts after several years, but the data herein is limited (9–11). Animal and human studies have demonstrated significant correlations between markers of NETosis and autoantibodies against beta-cell antigens (12), well in line with the hypothesis that NETs could be of relevance in the T1DM pathogenesis. Long-term diabetic complications, like micro-and macroangiopathy, have also been suggested to be related to NETosis (10, 13, 14).

There is growing evidence for the role of NETs in non-infectious conditions like autoimmune, metabolic, and cardiovascular diseases (15). NETosis has been extensively studied in the setting of atherosclerosis and atherothrombosis (16, 17) and seems to stimulate several mechanisms in atherogenesis, enhance thrombus formation and increase plaque vulnerability (18, 19). Clinical trials have lately demonstrated NETs markers to predict adverse cardiac outcomes in coronary artery disease (CAD) (20–22).

The exaggerated risk of CAD in long-term T1DM is well established (23, 24). Whether NETs are associated with diabetic CAD progression is unknown. We aimed to investigate whether levels of circulating NETs markers were elevated in long-term T1DM subjects compared to controls without diabetes, as well as explore whether levels of NETs were related to the presence of CAD.

## Materials and Methods

### Study Design and Participants

This is a sub-study of the Dialong study, which is a cross-sectional controlled study on long-term survivors of T1DM conducted in 2015 (25, 26). Patients were recruited from a specialized T1DM outpatients’ clinic, the Norwegian Diabetics’ Center (NDC) in Oslo, Norway. T1DM was clinically defined as a medical history characteristic of the disease, glycated hemoglobin (HbA1c) ≥ 48 mmol/mol (6.5%), and lack of insulin production as evidenced by a fasting c-peptide concentration <0.2 pmol/mL. All T1DM patients diagnosed before 1970 attending the NDC in 2015 were invited to participate and were asked to find participants for the control group by either asking their spouse or a close friend. Exclusion criteria herein were 1^st^-degree relatives and a known diagnosis of diabetes or HbA1c ≥ 48 mmol/mol (6.5%). All together 102 T1DM subjects and 75 controls were included. The regional ethics committee approved the study (project no. 2014/851), and the study conformed to the Declaration of Helsinki. All participants signed an informed consent.

Clinical data was collected from patient charts at NDC and a clinical examination during the first visit. All participants without established CAD were referred to computed tomography coronary angiography **(**CTCA) within a few months. Details of the CTCA procedure has been published earlier (26). Exclusion criteria for CTCA were eGFR <45 mL/min/1.73m^2^ or a fast irregular heart rate. All CT scans were analyzed by an experienced radiology consultant. The reports were reviewed and evaluated by a cardiologist for consideration of optimal medical treatment (OMT) or referral to invasive coronary angiography.

### Laboratory Methods

Blood samples were collected in fasting conditions between 08.00 and 10.30 at inclusion. Routine analyses were performed by conventional laboratory methods (Sysmex XN 9000, Sysmex Europe GmbH, Hamburg DE). Serum was prepared by centrifugation at room temperature within 1 h at 2500 g for 10 min for the analysis of citrullinated histone 3 (H3Cit) and PAD4, and EDTA plasma was prepared by centrifugation 2500 g for 20 min at 4°C for analyses of dsDNA and myeloperoxidase (MPO)-DNA. PAXGene Blood RNA tubes (Pre-Analytix GmbH, Hombrechtikon, Switzerland) were collected for RNA isolation. All materials were kept frozen at −80°C until further preparation and analysis.

dsDNA (ng/ml) was measured using Quant-iT™ PicoGreen^®^, a commercially available fluorescent nucleic acid stain. A standard calibration curve was prepared using thymus DNA of a known concentration, provided with the Quant-iT™ kit. PicoGreen^®^ was added to diluted EDTA plasma, and fluorescence was measured by use of Fluoroskan Ascent^®^ fluorometer, Thermo Fisher,Scientific Oy, Vantaa, Finland). DNase-free water was used for dilutions to minimise background fluorescence. The inter-assay coefficients of variation were 9.4%. The reproducibility in our lab has been validated in other and larger populations, with CVs ranging from 6.3% to 8.2% (6, 20, 21).

MPO-DNA was quantified by an in-house enzyme-linked immunosorbent assay (ELISA), as described by Kessenbroch at al (27). In brief, plates were coated and incubated overnight with the capture antibody anti-MPO (AbD Serotec, Hercules, CA, USA) and, after blocking with bovine serum albumin (BSA), patient plasma and a peroxidase-labeled anti-DNA antibody (Cell Death Detection Kit, Roche Diagnostics GmbH, Mannheim, Germany) were added. A peroxidase substrate was added after 2 hours of incubation, and after 40 min absorbance was measured as optical density (OD) units. The inter-assay coefficients of variation were 7.8%. The reproducibility in our lab has been validated with CVs ranging from 7.8% - 9.1% (6, 20, 21).

H3Cit and PAD4 were determined by commercially available ELISA kits (Cayman Chemical, Ann Arbor, MI, USA). The inter-assay coefficients of variation were 7.3% and 3.4%, respectively, which is accordance with the manufacturer’s indications.

For gene expression of PAD4, total RNA was isolated using the PAXGene Blood RNA kit (Qiagen GmbH for PreAnalytix, Hilden, Germany), with an extra cleaning step (RNeasy MinElute Cleanup kit; Qiagen), both according to the manufacturers’ instructions. RNA purity and quantity were determined by the Nano Drop™ 1000 Spectrophotometer (Thermo Scientific, Wilmington, DE, USA). Equal amount of total RNA per experiment (100 ng) was reversely transcribed into complementary DNA (cDNA) by the use of qScript cDNA SuperMix (Quanta BioSciences, Inc., Gaithersburg, USA). Expression of PAD4 mRNA was assessed by real-time polymerase chain reaction (RT-PCR) on the ViiA 7 Instrument (Applied Biosystems, by Life Technologies, Foster City CA, USA) using TaqMan Universal PCR Master Mix, No AmpErase UNG, and the PAD4 TaqMan assay (Hs01057483_m1). PAD4 mRNA levels were measured as relative quantification (RQ) (2^-ΔΔCt^ method) with beta-2-microglobulin (*β*_2_M) as the housekeeping gene (Assay ID Hs99999907_m1) (all Applied Biosystems).

### Clinical Outcome

Undiagnosed obstructive CAD was defined as the presence of a stenosis resulting in >50% lumen reduction in at least one coronary artery on CTCA. Absent CAD was defined as no plaque in any coronary artery on CTCA, while previous CAD was defined as either a previous episode of acute coronary syndrome, angina pectoris diagnosed by a cardiologist, or a previous revascularization procedure. Total CAD was defined as either previous CAD or obstructive CAD. We also defined three subgroups of coronary calcium score (CCS), based on previous reports (28): CCS 0, CCS 1-100, and CCS > 100.

### Statistical Analysis

The unpaired Student *t*-test and Mann–Whitney U test were used for group comparisons of continuous data with normal or skewed distributions, as appropriate. Categorical data were evaluated by the chi square test. The Kruskal–Wallis test was used to compare more than two groups. Correlation analyses were performed using Spearman’s rho. The significance level was set at p < 0.05. All analyses were performed using SPSS version 26 (IBM SPSS Inc., Armonk, NY: IBM Corp.).

## Results

### Characteristics of the Participants

Clinical and demographic characteristics of the study population are summarized in **Table 1**. There was no significant difference in leucocyte count or neutrophil count. The participants in the diabetes group had an average of 49 ± 4 years with T1DM, an average mean HbA1c during the follow-up time of 64 ± 8 mmol/mol, and an average current HbA1c of 57 ± 9 mmol/mol.

**Table 1:** Clinical and demographic characteristics of the study population.

	T1DM group (n = 102)	Control group (n = 75)	P
**Age**	61.9 ± 7.1	62.6 ± 7.0	0.517
**Sex, male**	51 (50)	34 (45)	0.54
**BMI kg/m^2^**	26.2 ± 4.0	27,5 ± 16.2	0.43
**sBP mmHg**	146.0 ± 19.6	137.2 ± 19.5	**0.004**
**dBP mmHg**	75.0 ± 8.3	81.2 ± 9.2	**<0.001**
**Current HbA1c mmol/mol**	57 ± 9	37 ± 3	**<0.001**
Mean HbA1c mmol/mol*	64 ± 8		
**Leukocyte count 10^9^/L**	6.4 ± 1.7	6.1 ± 2.2	0.284
**Neutrophil count 10^9^/L**	3.4 ± 1.2	3.3 ± 1.7	0.576
**CRP mg/L**	2.7 ± 3.9	2.2 ± 5.8	0.550
**Total cholesterol mmol/L**	5.0 ± 1.0	5.8 ± 1.2	**<0.001**
**HDL cholesterol mmol/L**	2.1 ± 0.6	1.7 ± 0.5	**<0.001**
**LDL cholesterol mmol/L**	2.7 ± 0.8	3.8 ± 0.8	**<0.001**
**TG mmol/L**	0.9 ± 0.4	1.1 ± 0.6	**0.003**
**Fasting glucose mmol/L**	8.6 ± 3.5	5.2 ± 0.6	**<0.001**
**Medications**			
**Statin (%)**	54 (54%)	9 (14%)	**<0.001**
**Aspirin (%)**	33 (33%)	8 (13%)	**0.004**
**Clopidogrel (%)**	1 (1%)	0	0,433
**Anticoagulation (%)**	5 (5%)	1 (1%)	0.195

Values are given as mean ( ± SD) or numbers (%), as appropriate. T1DM, type 1 diabetes mellitus; BMI, body mass index; sBP, systolic blood pressure; dBP, diastolic blood pressure; HbA1c, glycated hemoglobin; CRP, C-reactive protein; Tot, total; HDL, high density lipoprotein; LDL, low density lipoprotein; TG, triglyceride. *Based on the mean value during 30 years of measurements (25). Bold font indicates statistical significance.

### Levels of NETs Markers

Circulating dsDNA, MPO-DNA and H3Cit were available in all subjects (n=177), except one, whereas circulating PAD4 was available in 161 subjects due to lack of sample material. PAD4 gene expression was available in 169 subjects in which PaxGene tubes were available.

**Figure 1:**
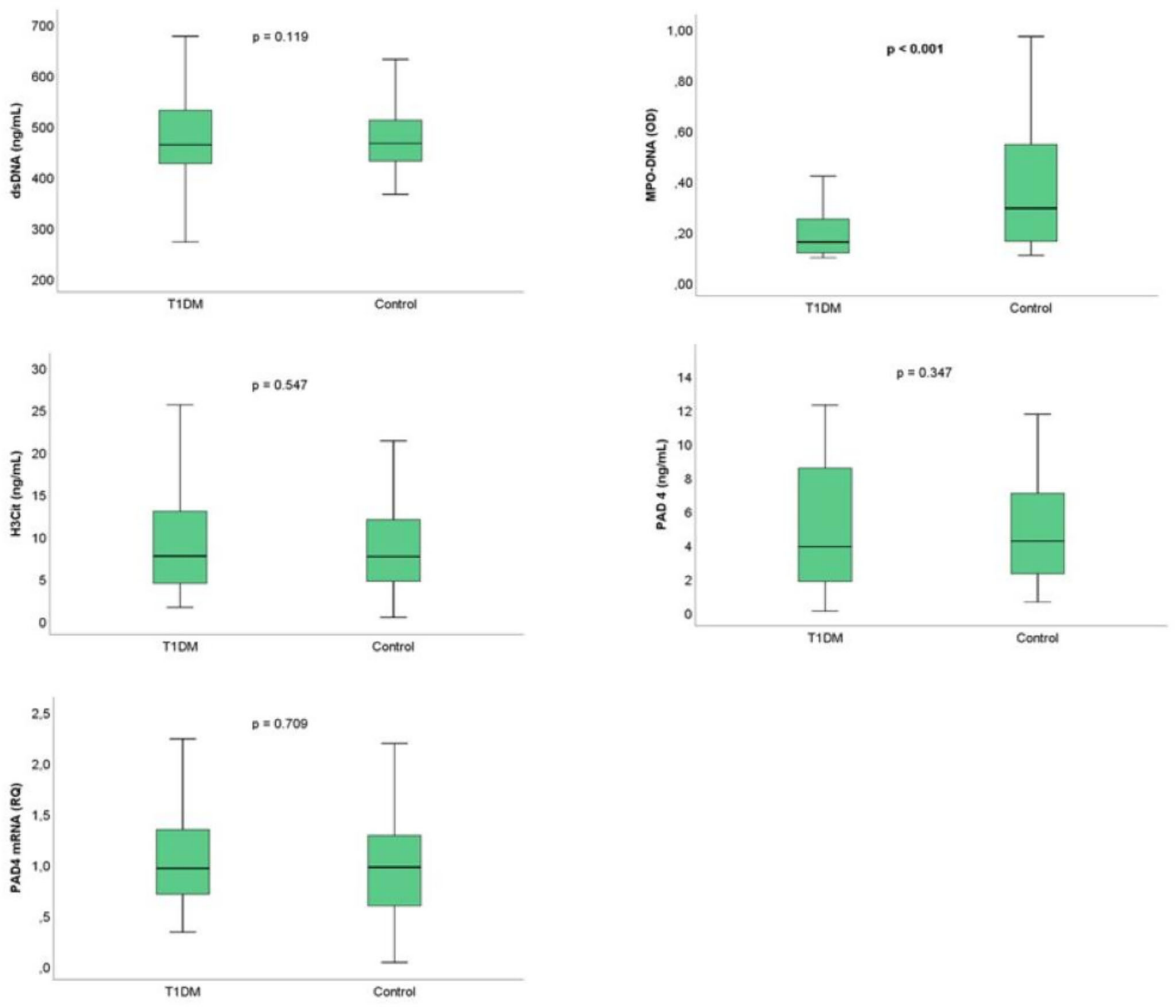
NETs marker levels according to presence of T1DM. Boxplot- The line inside the boxes represent the median, the box boundaries the 25^th^ and 75^th^ percentiles, while the lower and upper lines represent the 10^th^ and 90^th^ percentiles. T1DM, type 1 diabetes mellitus; dsDNA, double-stranded deoxyribonucleic acid; MPO-DNA, myeloperoxidase-DNA; H3Cit, citrullinated histone 3; PAD4, peptidylarginine deiminase 4.

Circulating MPO-DNA levels were significantly lower in patients with T1DM than in controls (0.17 vs 0.29 OD, p<0.001), while circulating dsDNA, H3Cit, PAD4 and gene expression of PAD4 did not differ with respect to the presence of T1DM (**Figure 1**).

**Figure 2:**
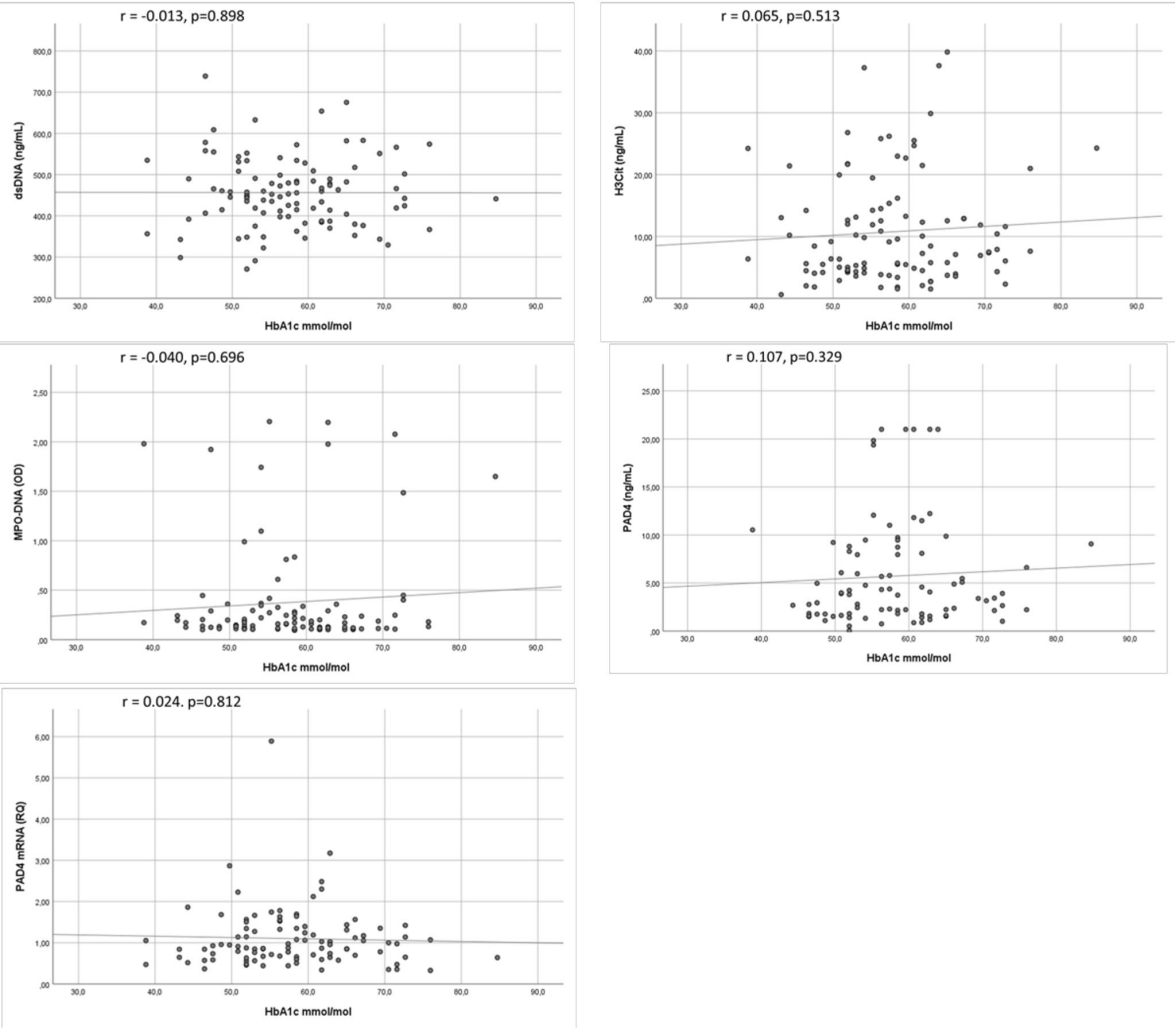
Correlations between NETs markers and current HbA1c in T1DM. Scatter graphs with trend line showing correlations between markers of NETs and HbA1c in TIDM. T1DM, type 1 diabetes mellitus; dsDNA, double-stranded deoxyribonucleic acid; MPO-DNA, myeloperoxidase-DNA; H3Cit, citrullinated histone 3; PAD4, peptidylarginine deiminase 4.

**Figure 3:**
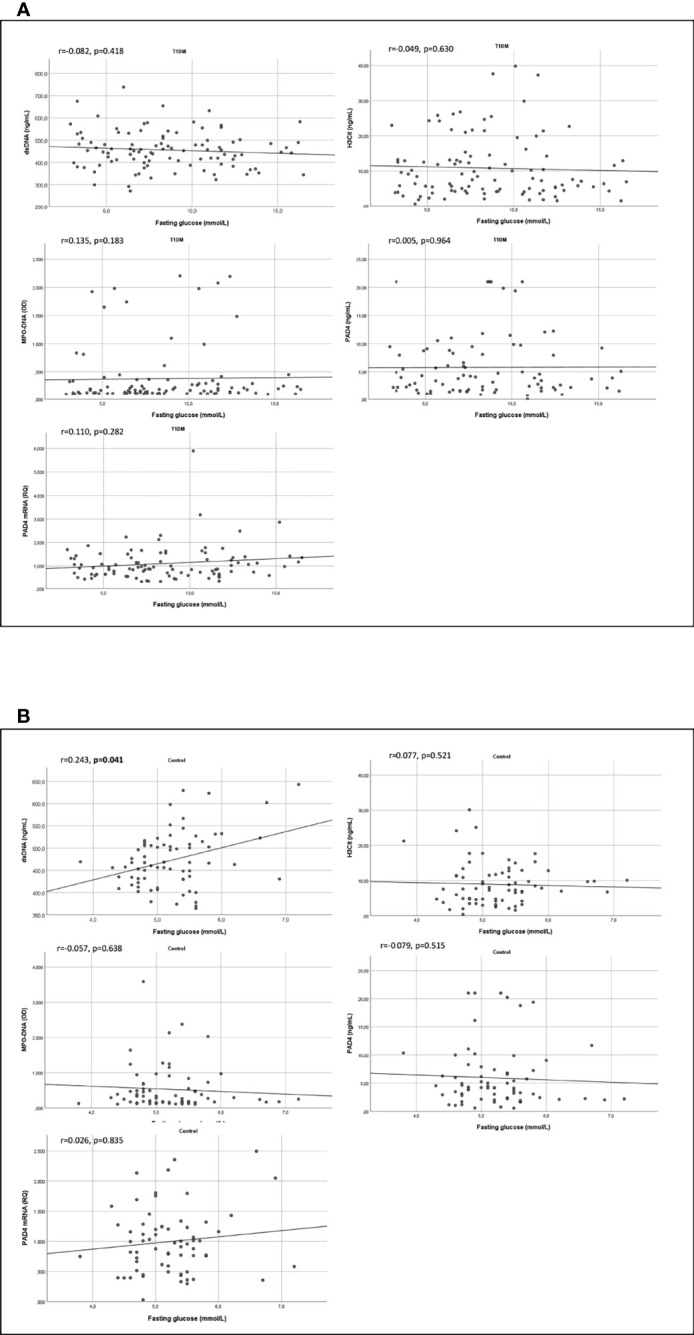
Correlations between NETs markers and fasting glucose in T1DM and controls. Scatter graphs with trend line showing correlations between markers of NETs and fasting glucose in TIDM **(A)** and in controls **(B)**. T1DM, type 1 diabetes mellitus; dsDNA, double-stranded deoxyribonucleic acid; MPO-DNA, myeloperoxidase-DNA; H3Cit, citrullinated histone 3; PAD4, peptidylarginine deiminase 4.

In the T1DM group, there were no significant correlations between current HbA1c and any NETs marker (r = -0.040-0.107, p = 0.513-0.898) (**Figure 2**). Then were neither no correlations between mean HbA1c and any NETs marker in the T1DM group (r=-0.083 – 0.077, p = 0.444-0.487). Also, when dividing current HbA1c in quartiles, no significant associations towards NETs markers were observed in the T1DM group (**Supplementary Figure 1**). Beyond for dsDNA in the control group (r = 0.243, p = 0.041), there were no significant correlations between fasting glucose and the NETs markers (**Figures 3** **A, B**).

### Levels of NETs Markers and CAD

The number of individuals with CAD in the T1DM and control group were 35 (34%) and 9 (12%), respectively. Circulating dsDNA, MPO-DNA, H3Cit and PAD4 did not differ according to the presence of CAD, neither in the T1DM nor in the control group (**Table 2**). H3Cit were non-significantly elevated in patients with T1DM and CAD compared to those without CAD. Gene expression of PAD4 were significantly higher in controls with CAD than in controls without CAD (**Table 2**).

**Table 2:** Levels of NETs markers according to the presence of CAD.

	dsDNA (ng/ml)	MPO-DNA (OD)	H3Cit (ng/ml)	PAD4 (ng/ml)	PAD4 mRNA (RQ)
**T1DM**					
**CAD+**	460 (411, 538)	0.16 (0.11, 0.28)	12.3 (5.4, 14.9)	4.0 (2.0, 9.5)	1.05 (0.85, 1.22)
**CAD -**	463 (430, 518)	0.19 (0.13, 0.36)	5.6 (4.2, 11.9)	3.7 (1.8, 8.3)	0.93 (0.63, 1.51)
**p**	0.846	0.325	0.071	0.445	0.232
**Control**					
**CAD+**	503 (429, 519)	0.18 (0.14, 0.84)	8.4 (3.3, 14.3)	4.6 (2.0, 9.5)	**1.32 (0.98, 1.97)**
**CAD -**	457 (429, 507)	0.33 (0.16, 0.55)	7.5 (4.7, 11.7)	4.2 (2.2, 6.6)	**0.88 (0.49, 1.24)**
**p**	0.491	0.529	0.854	0.792	**0.017**

Values are given as median (25, 75 percentiles). T1DM, type 1 diabetes mellitus; CAD, coronary artery disease; dsDNA, double-stranded deoxyribonucleic acid; MPO-DNA, myeloperoxidase-DNA; H3Cit, citrullinated histone 3; PAD4, peptidylarginine deiminase 4. Bold font indicates statistical significance.

**Figure 4:**
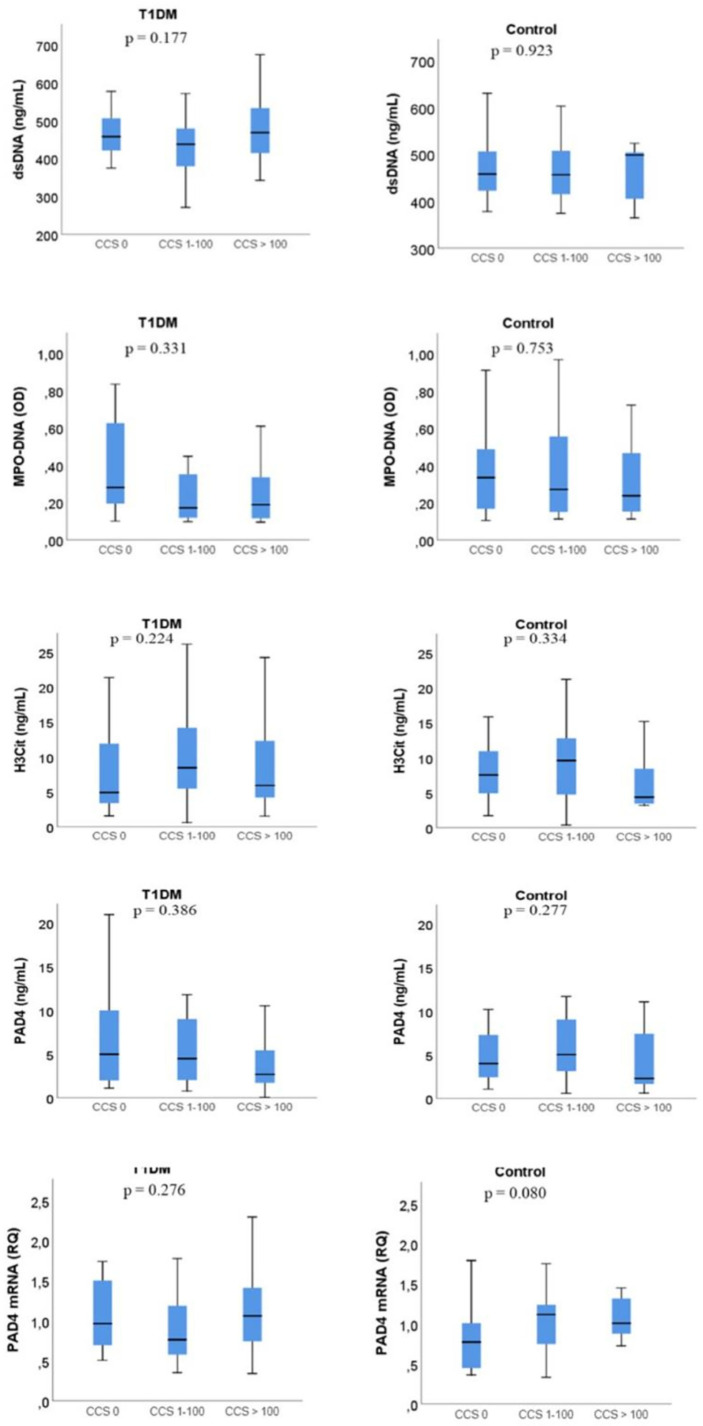
NETs marker levels according to coronary calcium score (CCS). Boxplot- The line inside the boxes represent the median, the box boundaries the 25th and 75th percentiles, while the lower and upper lines represent the 10th and 90th percentiles. T1DM, type 1 diabetes mellitus; dsDNA, double-stranded deoxyribonucleic acid; MPO-DNA, myeloperoxidase-DNA; H3Cit, citrullinated histone 3; PAD4, peptidylarginine deiminase 4. The p-value refer to difference between the three groups.

In the T1DM group, 12 (12%) had a CCS of 0, 29 (28%) had CCS 1-100, and 43 (42%) had CCS > 100. Equivalent numbers for the control group were 29 (39%), 21 (28%) and, 9 (12%), respectively. We did not find any significant differences between CCS subgroups and any NETs marker, neither in the total cohort nor in the groups separately (**Figure 4**). Correlation between CCS and the markers of NETs are included in supplementary materials (**Supplementary Figure 2**).

### Levels of NETs Markers and Neutrophil Cell Count

**Figure 5:**
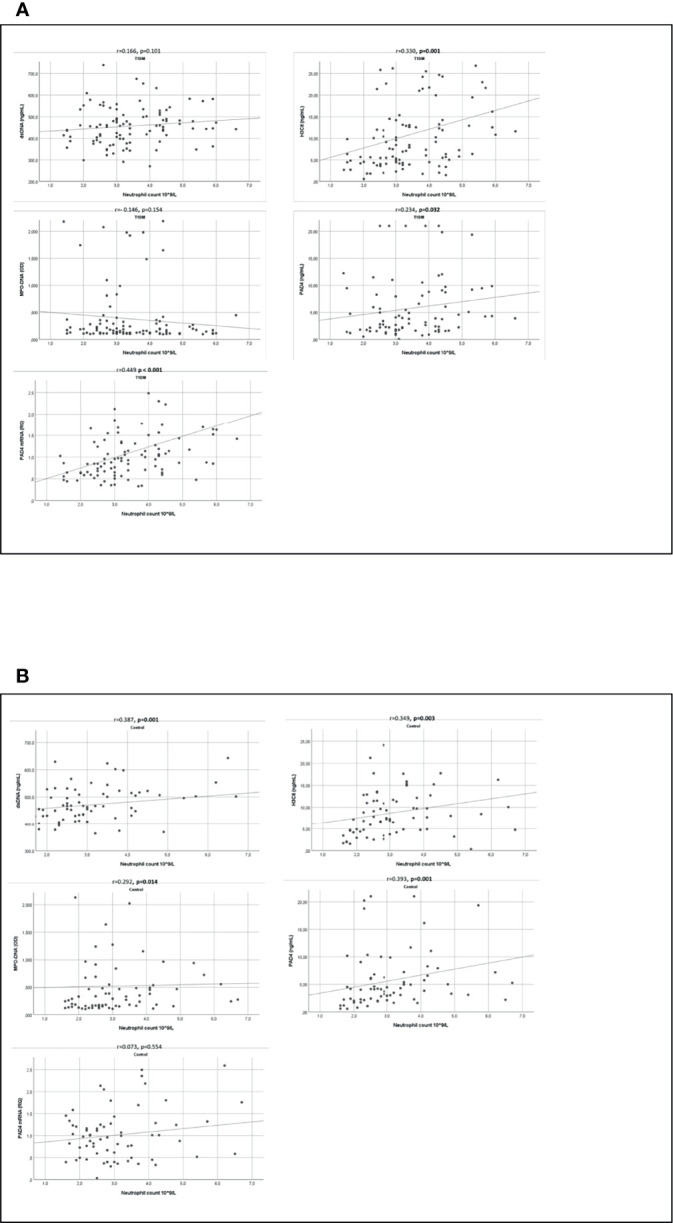
Correlation between NETs markers and neutrophil count. Scatter graphs with trend line showing correlations between markers of NETs and neutrophil count in TIDM **(A)** and controls **(B)**. T1DM, type 1 diabetes mellitus; dsDNA, double-stranded deoxyribonucleic acid; MPO-DNA, myeloperoxidase-DNA; H3Cit, citrullinated histone 3; PAD4, peptidylarginine deiminase 4.

While all circulating NETs markers correlated significantly with circulating neutrophils in the control group (r=0.292-393, p<0.014) (**Figure 5A**), only H3Cit and PAD4 correlated with neutrophils in the T1DM group (r= 0.330-0.449, p ≤ 0.001) (**Figure 5B**). The same pattern was observed for total leukocyte count (**Supplementary Table 1**).

### Intercorrelation Between Circulating PAD4 and Other NETs Markers

As a novel, unestablished circulating marker of NETs, *circulating* PAD4 were significantly correlated to MPO-DNA and H3Cit in the total population (r = 0.321, p < 0.001 and r = 0.599, p < 0.001, respectively) and in the groups separately (**Supplementary Figure 3**).

## Discussion

In this cross-sectional study of patients with long-term T1DM and age-matched controls, circulating levels of NETs markers were inconsistently associated with the presence of T1DM, shown by lower MPO-DNA levels in T1DM patients than in controls. NETs marker levels did not differ according to the presence of CAD. The circulating NETs markers were in general associated with neutrophil cell count in controls, but not in T1DM patients. Gene expression of PAD4 was associated with neutrophil cell count only in the T1DM group. Our results entail the possibility of altered neutrophil function and NETosis in T1DM.

Although hyperglycemia per se seems to increase NETs formation (5–8), previous studies on NETs in T1DM are inconsistent as to whether T1DM is associated with higher or lower NETs (10). Most studies assessing NETs markers in T1DM have been performed *at* the time of T1DM diagnosis or within the first few years (10). Markers of NETs in long-term T1DM have not previously been reported. Whether chronic hyperglycemia affects NETosis is unclear, although it could be hypothesized based on our findings. Also, the general lack of associations between neutrophil cell count and NETs markers, and the discrepancy in PAD4 gene expression and NETs markers in the T1DM group support this hypothesis.

The interplay between NETs and CAD is indisputable (19, 29). Still, to the best of our knowledge, NETs-related effects on CAD in patients with T1DM, either short- or long-term, have not been reported. We could not demonstrate any significant differences in circulating dsDNA, MPO-DNA, H3Cit, PAD4 and gene expression of PAD4 in accordance with the presence of CAD in the T1DM group. This might indicate that other mechanisms than NETosis is responsible for the accelerated atherosclerosis in T1DM.

A number of studies have assessed neutrophil function and count in T1DM, with diverging results (10). However, the current hypothesis is that patients with long-term T1DM most likely have some degree of neutrophil dysfunction (30–33). Whether this is due to the hyperglycemia per se, or a primary neutrophil defect is not known (31, 34). Given the lower levels of MPO-DNA in the T1DM group and overall absent link between neutrophil count and circulating NETs markers (except for H3cit and PAD4) in the T1DM group, our results are in line with a long-term T1DM mediated altered neutrophil function. Whether this indeed reduces NETosis is currently unclear.

Several markers are frequently used as surrogate markers of NETs (35). PAD4 activation is considered a crucial event during NETosis, yet protein levels of circulating PAD4 are not regarded as a traditional NETs marker. In our study, circulating PAD4 levels correlated with both circulating MPO-DNA and H3Cit. This indicate that circulating PAD4 levels could be a potential valuable marker of NETosis.

This study carries some limitations. CTCA is best for imaging of calcified coronary artery plaques, not for lipid-rich plaques prone to rupture and thrombosis. Although the technology is progressing rapidly, CTCA is currently not considered as the gold standard for diagnosing obstructive CAD. It should be emphasized that differences in the use of medications, serum lipids, and blood pressure may have masked the results. In particular, use of statins, aspirin, and P2Y12 inhibitors have all been shown to alter NETosis (36–38). There was significant more use of statin and aspirin the diabetic patients with CAD, compared to those without CAD. With the exception of MPO-DNA being lower in the patients with T1DM using statin (p=0.034), there was no difference in the NETs markers concerning the use of statins and aspirin in any group. The fact that the control group primarily consisted of spouses with similar environmental exposures might, e.g., by dietary habits, undervalue the effects of T1DM. Due to the relatively few participants, although many considering studying long-term T1DM, we cannot exclude type II errors. Due to few participants in the control group with CAD (n=9), it is difficult to conclude whether NETs are linked to CAD in this group.

In summary, in this cross-sectional study of patients with long-term T1DM and age-matched controls, circulating NETs levels were not consistently associated with the presence of T1DM or glycemic status. Markers of NETs did not differ according to the presence of CAD in patients with T1DM. Our results entail the possibility of altered neutrophil function and reduced NETosis in T1DM. This warrants further investigation.

## Data Availability Statement

The original contributions presented in the study are included in the article/**Supplementary Material**. Further inquiries can be directed to the corresponding author.

## Ethics Statement

The Norwegian Regional Committees for Medical and Health Research Ethics—South East (project no. 2014/851) approved the study. The patients/participants provided their written informed consent to participate in this study.

## Author Contributions

RH, TO, IS, and SA were central in the conception and design of this substudy, while IS, KH, and TB participated in the design of the original study. Analysis and interpretation of the data was performed by RH, TO, IS, and SA. SA prepared the initial draft, and all authors contributed substantively to review and editing of the manuscript. All authors have approved the final manuscript and have agreed to be accountable for the accuracy and integrity of the work.

## Funding

This work was supported by the Oslo Diabetes Research Centre and the Norwegian Diabetics’ Centre, as well as the Stein Erik Hagen Foundation for Clinical Heart Research, Oslo, Norway.

## Conflict of Interest

The authors declare that the research was conducted in the absence of any commercial or financial relationships that could be construed as a potential conflict of interest.

## Publisher’s Note

All claims expressed in this article are solely those of the authors and do not necessarily represent those of their affiliated organizations, or those of the publisher, the editors and the reviewers. Any product that may be evaluated in this article, or claim that may be made by its manufacturer, is not guaranteed or endorsed by the publisher.
