# Supplementary material for: NETosis in Long-Term Type 1 Diabetes Mellitus and Its Link to Coronary Artery Disease
Source: Front Immunol. 2022 Jan 5;12:799539. doi: 10.3389/fimmu.2021.799539 (PMC8767558; doi:10.3389/fimmu.2021.799539)
Supplement: Supplementary file 1 [file DataSheet_1.pdf]

Supplementary Material

Supplementary Table 1. Correlations between NETs markers and leukocyte count

		**dsDNA**	**MPO-DNA**	**H3Cit**	**PAD4**	**PAD4 mRNA**
**Leukocyte count**	T1DM group	0.178	-0.030	**0.365**	0.211	0.165
	p	0.074	0.768	**<0.001**	0.052	0.104
	Control group	**0.361**	**0.311**	**0.344**	**0.379**	-0.033
	p	**0.002**	**0.008**	**0.003**	**0.001**	0.790

Spearman's rank correlation coefficient (r). T1DM, type 1 diabetes mellitus; dsDNA, double-stranded deoxyribonucleic acid; MPO-DNA, myeloperoxidase-DNA; H3Cit, citrullinated histone 3; PAD4, peptidylarginine deiminase 4.

Figure Legends

Supplementary Figure 1- NETs marker levels according to HbA1c quartiles in T1DM

Boxplot The line inside the boxes represent the median, the box boundaries the 25th and 75th percentiles, while the lower and upper lines represent the 10th and 90th percentiles. T1DM, type 1 diabetes mellitus; HbA1c, glycated hemoglobin; dsDNA, double-stranded deoxyribonucleic acid; MPO-DNA, myeloperoxidase-DNA; H3Cit, citrullinated histone 3; PAD4, peptidylarginine deiminase 4.

Supplementary Figure 2. Correlations between CCS and markers of NETs in T1DM

Spearman's rank correlation coefficient (r) and scatter graphs with trend line showing relations between circulating markers of NETs. dsDNA, double-stranded deoxyribonucleic acid; MPO-DNA, myeloperoxidase-DNA; H3Cit, citrullinated histone 3; PAD4, peptidylarginine deiminase 4.

Supplementary Figure 3. Correlations between circulating PAD4 and other circulating markers of NETs in both groups together

Spearman's rank correlation coefficient (r) and scatter graphs with trend line showing relations between circulating markers of NETs. dsDNA, double-stranded deoxyribonucleic

acid; MPO-DNA, myeloperoxidase-DNA; H3Cit, citrullinated histone 3; PAD4, peptidylarginine deiminase 4.
