# Supplementary figures and images for: NETosis in Long-Term Type 1 Diabetes Mellitus and Its Link to Coronary Artery Disease
Source: Front Immunol. 2022 Jan 5;12:799539. doi: 10.3389/fimmu.2021.799539 (PMC8767558; doi:10.3389/fimmu.2021.799539)

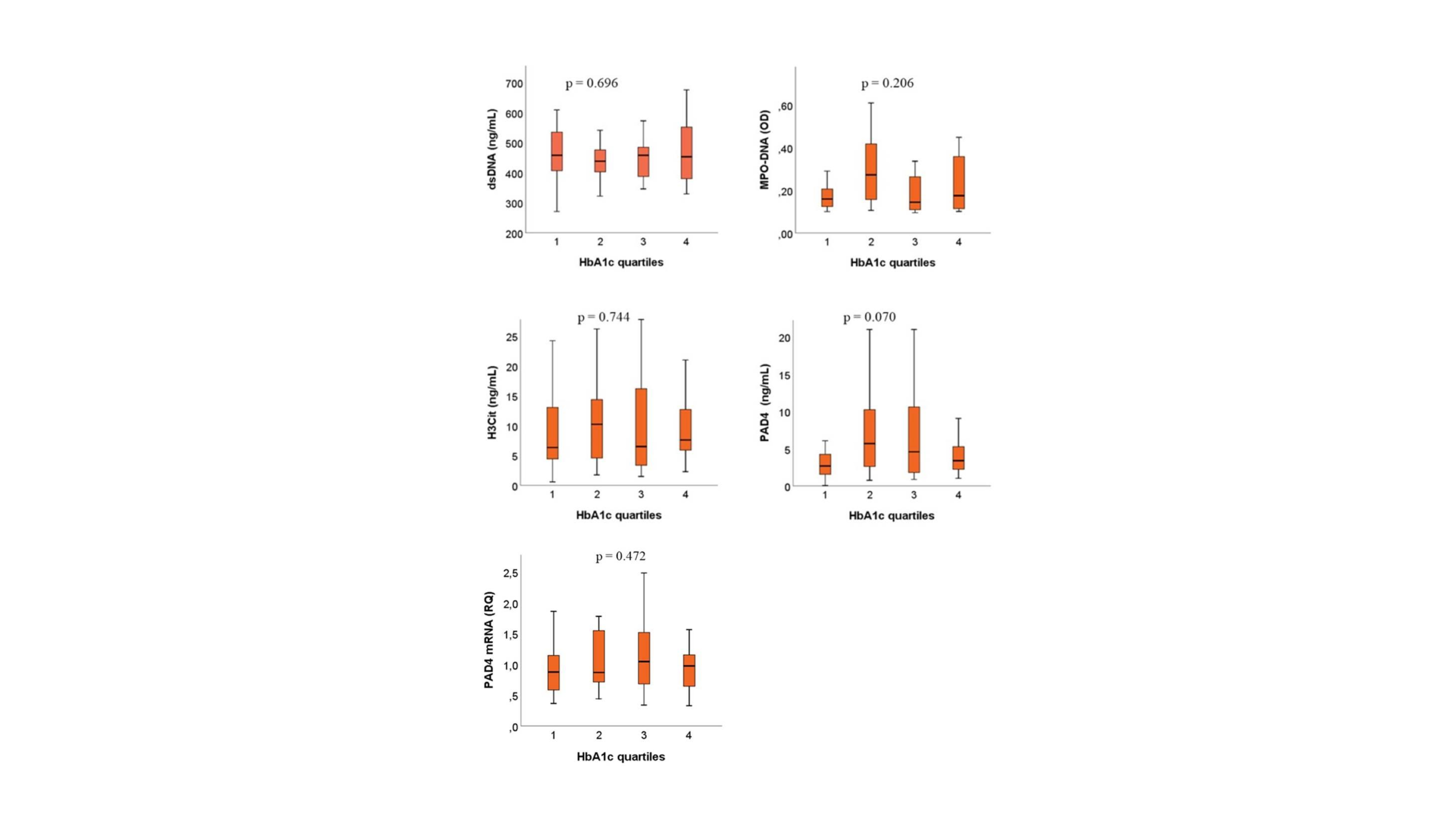

Supplement: Supplementary file 2 [file Image_1.jpeg]

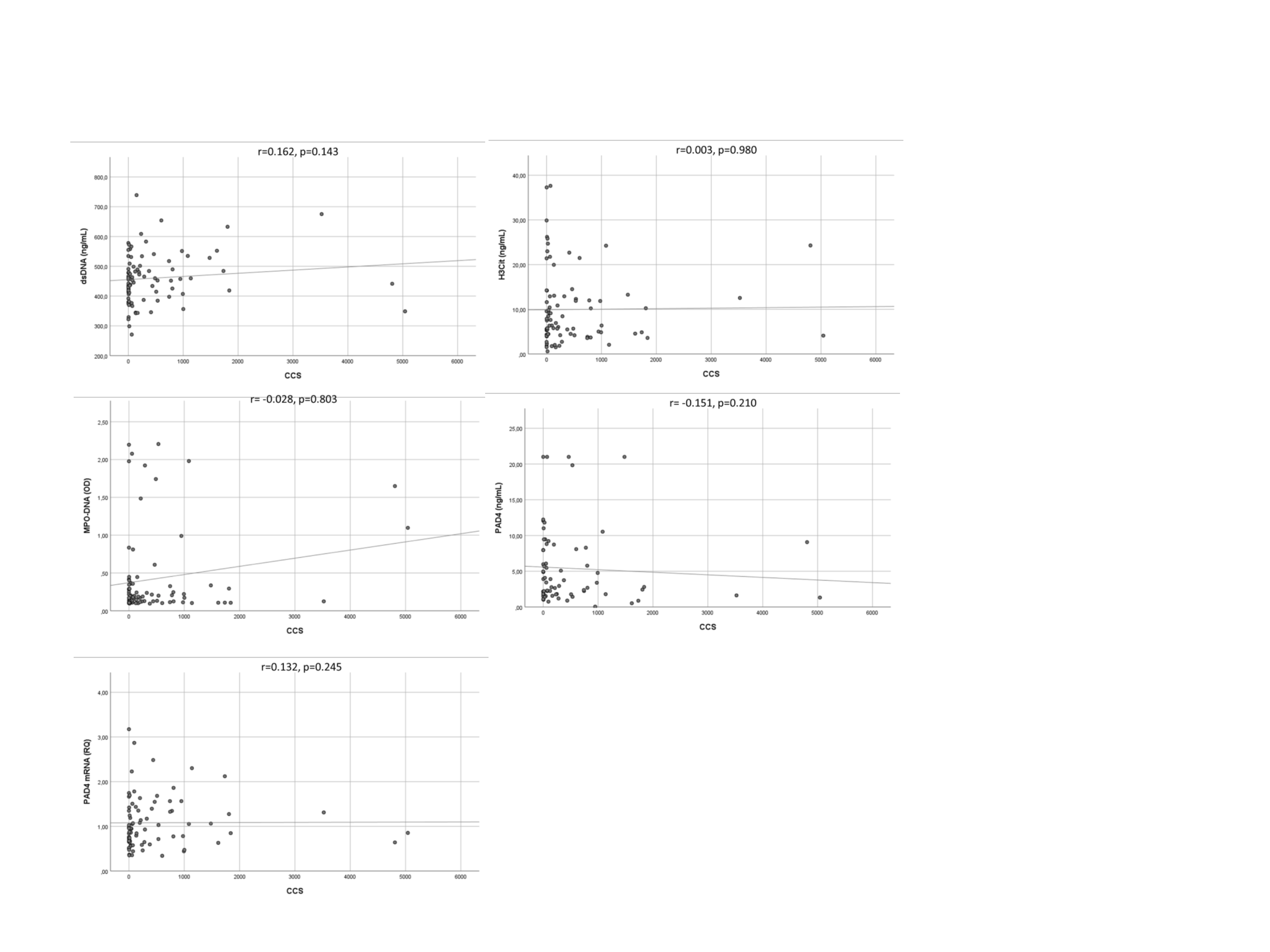

Supplement: Supplementary file 3 [file Image_2.jpeg]

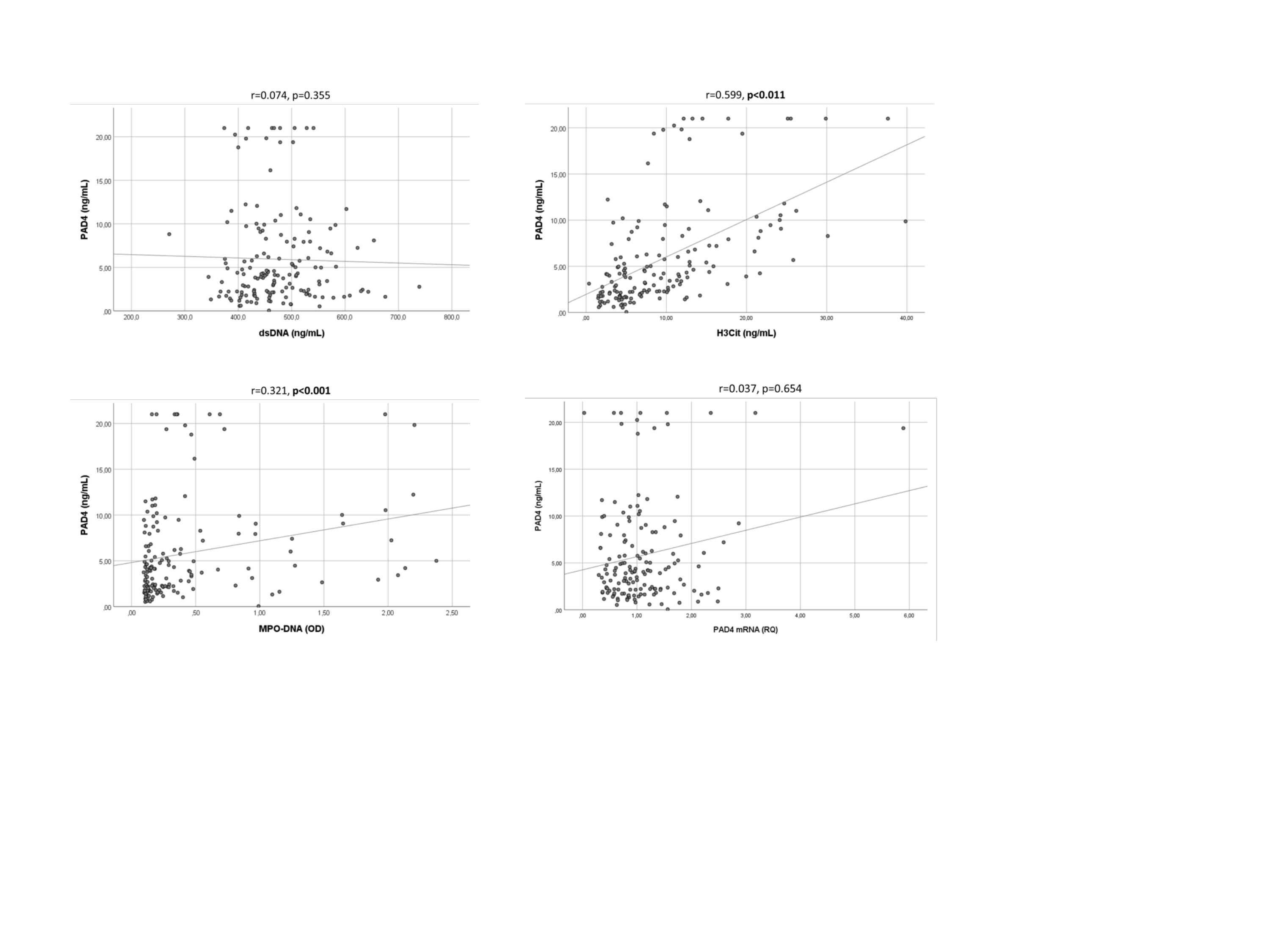

Supplement: Supplementary file 4 [file Image_3.jpeg]
